# Design and optimization of a Free-Space Optical (FSO) communication system for reliable outdoor connectivity in hospital departments in Malta

**DOI:** 10.12688/openreseurope.21257.4

**Published:** 2026-05-29

**Authors:** Ajay Sharma, Lalit Garg, Peter A. Xuereb, Ahmad Atieh

**Affiliations:** 1 Department of Computer Information Systems, Faculty of Information and Communication Technology, University of Malta, Msida, MSD2080, Malta; 2 Faculty of Commerce and Tourism, Industrial University of Ho Chi Minh City, Go Vap District, Ho Chi Minh City, Ho Chi Minh, Vietnam; 3 Optiwave Systems Inc., 7 Capella Court, Ottawa, Ontario, K2E 6A7, Canada

**Keywords:** Free-Space Optical Communication (FSO); Bit Error Rate (BER); Q-Factor; Optical Wireless Communication; Atmospheric Attenuation; EMI; Link Availability

## Abstract

**Background:**

Modern healthcare facilities face the challenge of ensuring secure, high-speed, and interference-free communication across hospital campuses. In Maltese hospitals, electromagnetic interference (EMI) from RF systems and the high cost of fibre deployment are major limitations. Free-Space Optical (FSO) communication offers a promising solution by providing gigabit-per-second transmission without EMI-related disruptions.

**Methods:**

This study proposes the design and parametric performance evaluation of a 1550 nm FSO communication system using OptiSystem 21 and MATLAB R2024b. The design incorporates Malta’s Mediterranean climate, dominated by haze with rare fog, into atmospheric attenuation models. System parameters, including attenuation coefficients, Q-factor, bit error rate (BER), and link availability, were evaluated under different weather conditions.

**Results:**

The system maintains reliable performance when rain attenuation is below 3.5 dB/km, achieving a Q-factor above 6 and error-free transmission in clear air with BER < 10 ^−12^ and a Q-factor of 13. The link operates within the explored simulation parameter space up to 2 km and sustains receiver sensitivity (−35 dBm) up to 5 km at 17 dBm transmit power. Simulations demonstrate high availability (>99%) under clear, hazy, and rainy conditions, while fog—occurring less than two days per year in Malta—reduces availability but does not impact the overall system feasibility. These availability estimates are modeled atmospheric predictions of a simulation.

**Conclusions:**

The developed standalone FSO system shows simulation viability of EMI-immune hospital connectivity in Maltese typical atmospheric models. Although the findings show high availability and great link margins, experimental validation and long-term performance evaluation would be needed to implement it in practice. The framework gives a hospital-based analytical foundation for the assessment of high-speed optical connections in the conditions of regional climate.

## Introduction

The healthcare sector requires fast, reliable, and secure communication links that prevent interference with medical instruments. The growing use of telemedicine protocols, high-resolution imaging, and electronic health records necessitates stringent EMI-free transmission requirements. The operation of critical medical equipment gets disrupted because traditional RF-based solutions cannot function effectively due to electromagnetic interference in sensitive healthcare settings. Fiber-optic base networks transmit high data rates with no interference. However, deploying them remains costly, and making changes to their setup proves difficult, particularly in active healthcare facilities. ^1^ ^,^ ^2^

Free-space optical (FSO) communication systems offer a promising alternative, combining low installation cost, EMI immunity, and gigabit-per-second performance. ^3^ The reliability and operational range of FSO links suffer degradation due to atmospheric attenuation caused by fog, haze, and rain. Real-world deployments need geographic and climatic adaptation for their successful implementation. This paper is concerned with feasibility assessment by simulation, assuming realistic Maltese climate conditions and not with experimental field implementation or with long-term operational validation.

The recent studies have pointed out the need to optimize Free-Space Optical (FSO) systems with regard to a variety of climate and application conditions. As an example, Sharma *et al*. ^4^ performed the evaluation of the efficiency of beyond 5G-related FSO connectivity, namely its stability in the presence of the haze effect in Malta, illustrating that 1550 nm is resilient with regard to the haze effect that reduces visibility. Likewise, Sharma *et al*. ^5^ compared the performance attenuation due to snow conditions using artificial neural networks to predict Bit Error Rate (BER), which indeed demonstrates AI-based models can be used to predict the performance of systems under unfavorable weather conditions. Previous experiments by Sharma and Kaler ^6^ handled inter-building high-speed connectivity using RF backup, with the practical advantages of hybrid designs being brought up in urban implementations. Recently, an overview on the role of Li-Fi in combination with the advent of IoT-based new sixth-generation (6G) communication systems has been provided by Sharma *et al*. ^7^ in their more recent and expanded capabilities of optical wireless links and networks beyond the well-known FSO. These studies establish a foundation for climate-aware and application-specific optical wireless networks, motivating the present research.

The Mediterranean climate of Malta features clear skies and moderate rainfall, while the winters tend to be dominated by haze and fog that occurs very rarely. ^8^ The FSO system deployment in such conditions is possible if engineers focus on building resilient systems against haze and rain. This research work develops a 1550 nm FSO system for Maltese hospital campuses that incorporates environmental modeling, addresses medical requirements, and designs high-reliability optical links.

In this work, optimization is not done with heuristic or machine learning based optimization algorithms, but is a systematic tuning and checking of parameters when varying the link distance, attenuation, transmitter power and receiver sensitivity within the simulated FSO design space.

**Figure 1.**
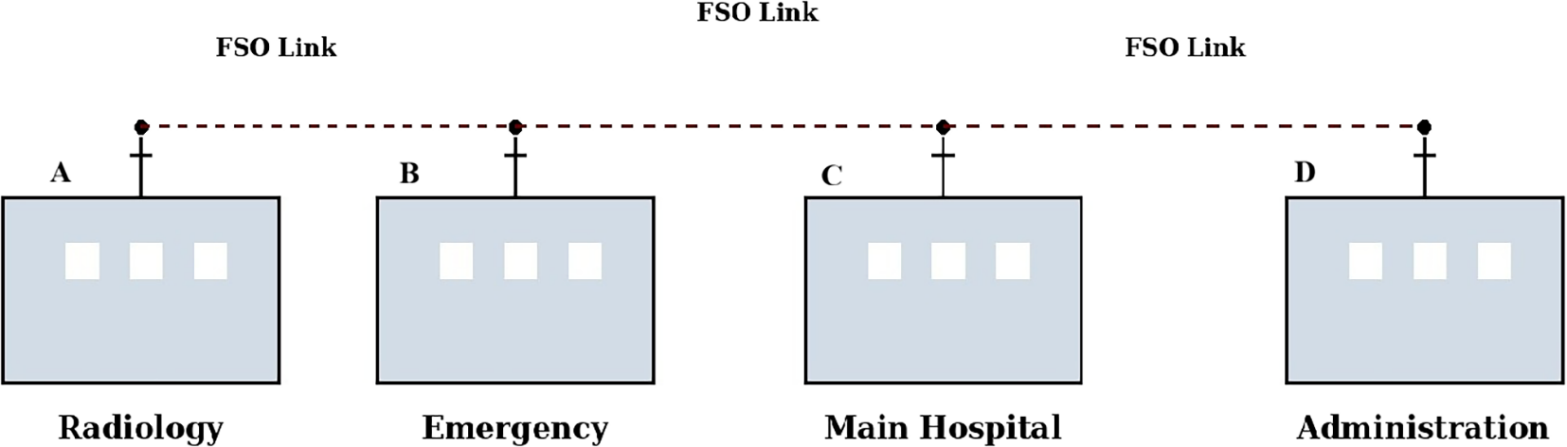
Conceptual FSO-based interdepartmental hospital connectivity model showing symbolic communication links between hospital departments. ^6^

This work proposes a standalone 1550 nm FSO system designed for Maltese hospital campuses, where EMI-safe, cost-effective, and high-speed communication is essential. Unlike conventional RF-based networks, the proposed system eliminate risks associated with EMI and remove reliance on physical fibre network using wireless optical links under modeled conditions. The figure below, in Figure 1, presents a conceptual view of free-space optical (FSO) links connecting hospital departments at the campus level through EMI-free high-speed communication without using physical cables.

A conceptualized view of the possible interdepartmental communication links on a hospital campus is shown in Figure 1. The modeled scenario is that the Radiology Block is a transmitter node (A), and the Main Hospital Block is a receiver node (C). There may also be other communication circuits between the Emergency (B) and Administration (D) departments, as required by operational needs. The figure illustrates optical wireless connectivity between buildings but does not specify the network topology.

The simulated link distances used in this study are in the range of 0.5 km to 2 km, which is deemed a realistic deployment scenario for hospitals over campus. In a good atmosphere, single-hop communications over longer distances might be possible within reasonable attenuation limits. But in bad weather (like fog), shorter multi-hop interconnections might offer better link reliability and lower attenuation buildup.

Although the given results point to the high expected availability in the set of atmospheric conditions that the model presupposes, the practical implementation can involve additional experiments and long-term tests.

## Related work and research gaps

Different research studies have been conducted to optimize the operation of FSO systems under different climate conditions. A detailed survey focusing on turbulence impacts in FSO links is presented in Khalighi and Uysal, ^1^ together with channel models developed by Ghassemlooy *et al*. ^9^ for signal attenuation prediction using MATLAB software. The authors of Al-Gailani *et al*. ^10^ analyzed drone-based medical logistics through FSO systems, and Gupta *et al*. ^11^ developed machine learning techniques to enhance urban FSO deployment stability. Awan *et al*. ^12^ investigated the FSO system for desert conditions, and the research by Habaebi *et al*. ^13^ studied tropical environments, confirming that the 1550 nm wavelength outperforms 850 nm by reducing scattering under haze and rain. Ahmed *et al*. ^14^ conducted healthcare research to discover wireless communication needs for future hospital networks, which required high-speed performance and EMI-free and reliable connectivity. Other works proposed hybrid FSO/RF architectures to mitigate fog disruptions; however, these solutions introduce unnecessary complexity and higher costs in regions with very rare fog, such as Malta (occurring fewer than two days per year). ^8^ Esmail *et al*. ^15^ presented models of fog attenuation to evaluate FSO link reliability under low-visibility scenarios.

Despite these advances, a critical gap remains: exploiting FSO systems for EMI-sensitive hospital environments has not been fully addressed. Current models either focus on general climatic effects or propose hybrid designs that are not cost-effective in low-fog regions. Furthermore, system designs tailored to specific regulatory requirements (IEC 60601–1-2) and regional climates like Malta are largely missing.

This study addresses these gaps by proposing a standalone FSO communication framework optimized for hospital applications in Malta’s Mediterranean climate. The design ensures reliable inter-departmental connectivity, compliance with EMI standards, and practical deployment without the overhead of hybrid architectures.

## Contributions and novelty

This paper proposes a Free-Space Optical (FSO) communication system specifically designed and parametrically evaluated for Maltese hospital environments, where EMI-free, cost-effective, and high-speed connectivity is essential. The novelty of this work lies in combining climate-optimized modeling, wavelength selection, and hospital-specific requirements into a single simulation-based analytical framework. Local meteorological data, including haze visibility of around 5 km and worst-case rain attenuation of 3.5 dB/km, are incorporated into MATLAB-based simulations to refine attenuation models and provide accurate performance predictions under Mediterranean conditions. The system employs a 1550 nm wavelength, which prior studies ^16^ have shown to be superior in hazy atmospheres compared to shorter wavelengths such as 785 nm, making it a technically appropriate choice under the modeled Maltese climate conditions, where haze is common and fog is extremely rare. Unlike hybrid FSO/RF designs, the simulations suggest that an isolated FSO design might be technically possible in Malta in the conditions of attenuation simulated; deployment-level resiliency and redundancy would have to be subject to additional field testing. The design is tailored to mission-critical hospital communication, enabling real-time telemetry, secure transmission of medical imaging, and electronic health records while fully complying with EMI safety standards (IEC 60601–1-2). ^17^ The research thus provides a locally-specific simulation model of assessing EMI-conformant and climate-sensitive FSO hospital links, and forecasted availability of 99% during clear and moderate weather. These are estimates of analytical performance and not deployment certifiable guarantees.

## Methods

### Mathematical modelling of the proposed FSO system

This section derives the mathematical framework governing the performance of the proposed 1550 nm FSO system, aligning with system parameters chosen for Malta’s weather conditions and referencing prior works for each critical equation. Optimization in this paper is the systematic parametric analysis over a set of predetermined transmitter power, attenuation, and link-distance space instead of the use of a formal global optimization algorithm. The configuration that produces the largest link margin within the constraints of the Maltese climate is therefore analytically preferable in the parameter space that is explored.

***Transmitter model***

The transmitter operates at 1550 nm with an optical power of 50 mW (extinction ratio 20 dB). It includes a laser diode modulated by a Mach-Zehnder modulator (MZM). A sequence of random bits is encoded on non-return-to-zero electrical pulses, which modulate the laser through the MZM. The MZM outputs a total optical power as follows:

$$P_{total}=50\;mw\;(17\;dBm)$$ (1)

The extinction ratio ER is defined as 9:

$$ER=10.log_{10}\left(\frac{P_{1}}{P_{0}}\right)=20\;dB$$ (2)

***FSO channel model***

The free-space optical channel accounts for both atmospheric attenuation and geometric loss.

**Atmospheric Attenuation:** Using Kim’s model, ^9^ the optical signal attenuation *α*( *λ*) is calculated as:

$$\alpha (\lambda )=\frac{3.91}{V}{\left(\frac{\lambda }{550}\right)}^{-q}$$ (3)

Where *λ* = 1550 nm, *V*: visibility (km), *q* = Particle size-dependent coefficient.

For Malta’s climate, representative attenuation values are: clear air → α ≈ 0.43 dB/km (V = 10 km, q = 1.3); haze → α ≈ 2.7 dB/km (V = 5 km, q = 1.6); fog → α ≈ 15 dB/km (V = 1 km, q = 0.585·V + 1.05); rain → α = 3.5 dB/km (moderate rain). ^18^ These values reflect typical conditions in Malta’s Mediterranean environment.

**Beam Divergence and Transmitter Aperture:** The transmitter’s beam divergence ( *θ*_*tx*_ = 1.5 mrad) and transmitter aperture diameter ( *D*_*tx*_ = 10 cm) determine the initial beam waist *W*_0_ ^9^:

$$\;W_{0}=\frac{D_{tx}}{2}=5\;cm$$ (4)

**Geometric Loss:** The Geometric loss *L _g_* arising from beam spreading, is modelled as 9:

$$L_{g}=10.log_{10}\left({\left(\frac{\theta_{tx}.L}{D_{rx}}\right)}^{2}\right)$$ (5)

Where: L = 1 km; *D*_*rx*_ = 20 cm (receiver aperture) producing

$$L_{g}=10.log_{10}\left({\left(\frac{1.5\times 10^{-3}.1000}{0.2}\right)}^{2}\right)=11.25\;dB$$ (6)

**Total Channel Loss:** The total channel loss combines atmospheric and geometric components ^9^:

$$L_{total}=\alpha (\lambda ).L+L_{g}$$ (7)

For instance, under a *fog* condition (α = 15 dB/km) over 1 km:

$$L_{total}=(15\times 1)+11.25=26.25\;dB$$ (8)

For *clear air* conditions (α = 0.43 dB/km) over 1 km, the total loss would be ≈0.43 + 11.25 = 11.68 dB.

***Receiver model***

A PIN photodiode at the receiver converts the optical signal to electrical current, with performance governed by the photodiode’s responsivity and noise characteristics. ^9^

**Received Optical Power:** The received optical power *P*_*r*_ at the photodiode can be expressed as:

$$P_{r}=P_{total}-L_{total}$$ (9)

where all values are in linear scale or dB as appropriate. For example, under clear-air conditions over 1 km L _total_ = 0.43 + 11.25 = 11.68 dB:

$$P_{r}=17\;dBm-11.68\;dBm=5.32\;dBm\;(3.4\;mW)$$ (10)

**Photodiode Output Current:** The photocurrent generated *I*_*ph*_ is given by 9:

$$I_{ph}=R.P_{r}+I_{d}$$ (11)

where *R* is the photodiode responsivity (A/W) and *I*_*d*_ is dark current. Given a typical PIN photodiode with *R* = 0.8 A/W and a negligible dark current *I*_*d*_ = 10 nA. The photocurrent for *P*_*r*_ = 3.4 mW is:

$$I_{ph}=0.8\times 3.4\times 10^{-3}+10\times 10^{-9}=2.72\;mA$$ (12)

**Signal-to-Noise Ratio (SNR):** The received SNR is affected by noise sources dominated by the shot noise $\sigma_{\text{shot}}^{2}$ and thermal noise $\sigma_{\text{thermal}}^{2}$ . ^9^

Shot Noise Variance Calculation

$$\sigma_{shot}^{2}=2q\left(I_{ph}+I_{d}\right)\;B=2.(1.6\times 10^{-19}).(2.72\times 10^{-3}).(1.25\times 10^{9})=1.088\times 10^{-12}A^{2}$$ (13)

Thermal Noise Variance Calculation

$$\sigma_{thermal}^{2}=\frac{4KTB}{R_{L}}=\frac{4.(1.38\times 10^{-23}).300.(1.25\times 10^{-9})}{50}=4.14\times 10^{-13}A^{2}$$ (14)

Assuming a receiver bandwidth of B = 1.25 GHz, at room temperature T = 300 K, and load resistance R _L_ = 50 Ω.

The SNR is given by:

$$SNR=\frac{I_{ph}^{2}}{\sigma_{shot}^{2}+\sigma_{thermal}^{2}}$$ (15)

SNR (dB) ≈ 66.93 dB.

**SNR Validation**: The calculated SNR (66.93 dB) aligns with the simulated OSNR of 51.64 dB, accounting for optical vs. electrical domain differences.

**Relation between Q-Factor and BER**

The theoretical relationship between Q-factor and BER in optical communication is given by $BER=\frac{1}{2}erfc\left(\frac{Q}{\sqrt{2}}\right)$ . High *Q* values result in extremely small BER values (for example, *Q* = 13 yields BER < 10 ^−30^), which cannot be measured in practice. To avoid reporting unrealistic values, all BER values below 10 ^−12^ are reported as “BER < 10 ^−12^”, representing the practical error-free region.

***Link budget validation***

The link budget confirms feasibility under Malta’s weather conditions. ^5^ Critically, receiver sensitivity depends on the bit rate due to noise bandwidth constraints. For the PIN photodiode, sensitivity is calculated as follows:

**Receiver Sensitivity vs. Bit Rate:** The minimum detectable power *P _min_* (sensitivity) for a target BER (e.g., 10 ^−9^) scales with bit rate *R _b_* ^19^

$$P_{min}\;\alpha \;Q.\sqrt{R_{b}.N_{0}}$$ (16)

Where: Q = 6 (BER = 10 ^−9^) and N _0_ = Noise spectral density (W/Hz).

For the *R _b_* = 1.25 Gbps NRZ system: Sensitivity = −35 dBm. ^20^

So received power *P _r_* = 5.32 dBm exceeds by 40.32 dB, so the 40.32 dB margin ensures robustness even at higher bit rate performance.

### System design and simulation setup

The developed FSO communication system is designed to support high-performance connectivity between buildings in Maltese hospital campuses despite regionally observed haze, seasonal rainfall, and infrequent fog. The system architecture was modeled and simulated using OptiSystem, with atmospheric attenuation values derived from measured climate data processed in MATLAB. All simulations were performed using OptiSystem 21 (licensed at the University of Malta) and MATLAB R2024b. Authentic weather statistics were obtained from the Malta Meteorological Office and National Statistics Office (NSO) climate reports, ^8^ ^,^ ^21^ including visibility and precipitation data for 2020–2023. These data were processed via the Kim attenuation model to yield realistic attenuation coefficients representative of Malta’s climate (e.g., 0.43 dB/km for clear air, 2.7 dB/km for haze, 3.5 dB/km for moderate rain, and an extreme 25 dB/km for dense fog used as a safety margin). A wavelength-division multiplexing (WDM) transmitter operating at 1550 nm was modeled in OptiSystem with an output power of 50 mW. The 1550 nm wavelength was chosen for its low atmospheric attenuation, resilience against haze, and compatibility with commercial optics at the 1.25 Gbps data rate needed for hospital applications (patient monitoring, MRI image transfer, large file backup, etc.). The transmitter can be wavelength-tuned within the C-band (around 1500–1600 nm) if required for system flexibility.

The free-space channel was modeled for a nominal 1 km link, representing typical inter-building distances on a hospital campus. For simulations, an extremely high attenuation case of 25 dB/km was included to represent worst-case fog (far exceeding typical Maltese conditions) as a stress test. More realistic attenuation values were calculated in MATLAB for clear air (0.43 dB/km), haze (2.7 dB/km), and rain (3.5 dB/km), as noted above. The channel assumes a beam divergence of ~1.5 mrad and a receiver aperture of 20 cm, which together produce a geometric loss of about 11.25 dB for a 1 km link. Malta’s stable weather and low wind environment allow for passive rooftop transceiver mounting with minimal misalignment over time, reducing the need for active alignment systems.

On the receiver side, a PIN photodiode module with ~0.94 GHz electrical bandwidth was selected to minimize inter-symbol interference, ensuring proper demodulation even during low-visibility periods. Eye diagram analysis in simulation provides key indicators of signal integrity: for instance, the eye height is 1.88985e ^−6^ and the decision threshold 1.14616e ^−6^. Under clear-air conditions, these values correspond to a wide eye opening and stable signal detection. The system is designed with a sufficient power margin (≈25 dB/km beyond clear-air attenuation) to reliably handle rare fog events (which occur only ~2 days per year in Malta). This makes implementation simpler and more cost-effective than complex hybrid FSO/RF designs, as the FSO link alone can tolerate those rare extremes through power control or temporarily reduced data rate if needed.

All the simulation parameters which are the extinction ratio (20 dB), transmitter aperture (10 cm), beam divergence (1.5 mrad), receiver aperture (20 cm), photodiode responsivity (0.8 A/W), receiver bandwidth (0.94 GHz), and noise temperature (300 K) are directly given so that they can be easily replicated using the OptiSystem or other similar numerical solvers. Attenuation and link budget calculations using MATLAB are a direct replication of the equations in this section, which makes the methodology straightforward to understand.

**Figure 2.**
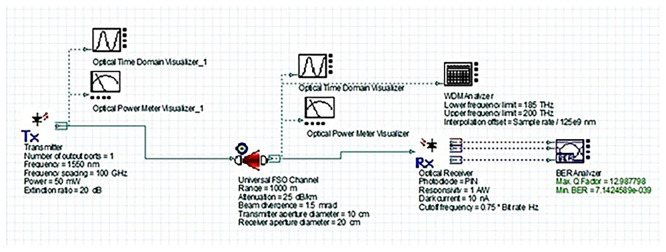
OptiSystem simulation layout of the proposed 1550 nm FSO system.

Figure 2 shows the simulated FSO system layout. It includes a 1550 nm WDM transmitter with a high-power laser, a universal FSO channel model incorporating realistic atmospheric attenuation conditions, and a PIN photodiode-based receiver. The depicted FSO link range represents the maximum distance between hospital buildings in the campus scenario (on the order of 1–2 km for core departments).

The Mediterranean climate of Malta consists of high humidity combined with coastal haze and very rare fog, and sporadic rainfall. ^22^ A summary of the studied weather conditions and their effects on the FSO link is provided in Table 1. These conditions form the basis for the attenuation values used in the simulations.

**Table 1.** Selected Weather Conditions for Malta’s FSO System Simulation.

Weather Condition	Visibility (km)	Attenuation (dB/km)	Impact on FSO
Clear Air	> 10	0.2–0.5	Minimal signal loss
Moderate Haze	2–5	1.5–3.0	Increased scattering in humid coastal zones
Moderate Fog	0.5–1	8.0–15.0	Severe attenuation; occurs <2 days/year
Moderate Rain	2–5	1.0–3.5	Absorption and scattering reduce signal reliability
Thick Cloud	—	—	Not applicable (links below cloud layer)
Snow	—	—	Not applicable (snow not observed in Malta)

In parallel, climate data was analyzed to understand the frequency of various weather scenarios. Table 2 summarizes the annual frequency of clear air, haze, rain, and fog in Malta (based on 2020–2023 data), along with the dominant seasons for each condition. ^21^ As shown, clear-air conditions dominate (~68% of the time), followed by moderate haze (~25%). Moderate rain is relatively infrequent (~6%), and significant fog is extremely rare (<1%). These statistics validate the decision to prioritize clear-air, haze, and rain scenarios in the system’s attenuation modeling and performance testing. The rarity of fog supports using a simplified standalone FSO system without the need for hybrid RF redundancy in most cases.

**Table 2.** Climate Conditions and Link Scenario Breakdown. ^21^

Weather Condition	Visibility (km)	Annual Frequency	Dominant Seasons
Clear Air	> 10	68%	Summer
Moderate Haze	2–5	25%	Winter/Spring
Moderate Rain	2–5	6%	Autumn
Moderate Fog	0.5–1	< 1%	Winter mornings

The aim of the present study is mainly dedicated to the deterministic atmospheric attenuation model in the Maltese climate. The present form of this work does not explicitly model advanced stochastic effects like atmospheric turbulence, scintillation, beam wander and beam pointing errors. These factors are also likely to impact the long-distance FSO operation under dynamic atmospheric conditions and will be explored in future research via statistical and experimental studies.

## Results and discussion

A detailed simulation of the proposed system using both OptiSystem and MATLAB validates its **simulation-based feasibility under modeled atmospheric conditions representative of Maltese hospital campuses**. The performance analysis of the proposed FSO system specifically considers Malta’s climate, ^1^ where haze and seasonal rainfall are more prevalent than fog. The assessments of signal integrity and operational feasibility are based on four key performance metrics: BER, Q-Factor, link availability, and maximum reliable distance. Table 1 presents the operational impact of various attenuation levels on the outdoor hospital system functionality. In the simulations, 1.25 Gbps NRZ pulses are transmitted through the FSO channel, representing typical data rates required for hospital applications such as real-time patient monitoring, diagnostic imaging transfer, and secure medical record exchange.

### BER performance and eye diagram analysis

**Figure 3.**
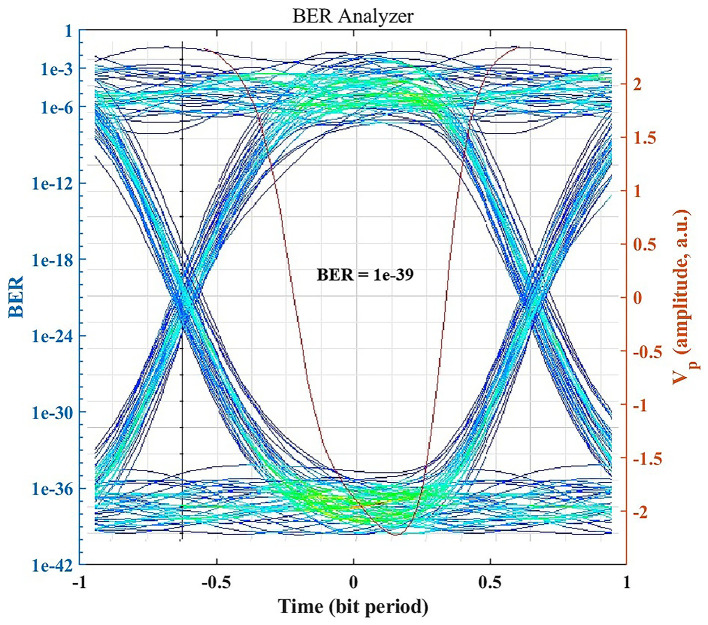
Eye diagram and BER under clear-air conditions.

Under standard clear-air conditions, the OptiSystem BER Analyzer indicates error-free performance. Figure 3 shows the eye diagram for the received signal in clear air, which exhibits a wide eye opening and stable timing. The simulated BER reaches the practical error-free region (BER < 10 ^−12^, which lies below commonly adopted analytical thresholds for optical communication reliability. Values below 10 ^−12^ are theoretical and represent the error-free operating region rather than physically measurable BER values. The clear eye pattern and minimal inter-symbol interference confirm that the system can sustain high-integrity, gigabit-per-second data transmission in favorable weather, within the modeled channel assumptions.

In practical optical communication systems, BER values below 10 ^−12^ are commonly regarded as representing near error-free transmission performance because further BER reductions have minimal practical impact on communication reliability. ^23^

### Signal Integrity Assessment through Q-Factor

**Figure 4.**
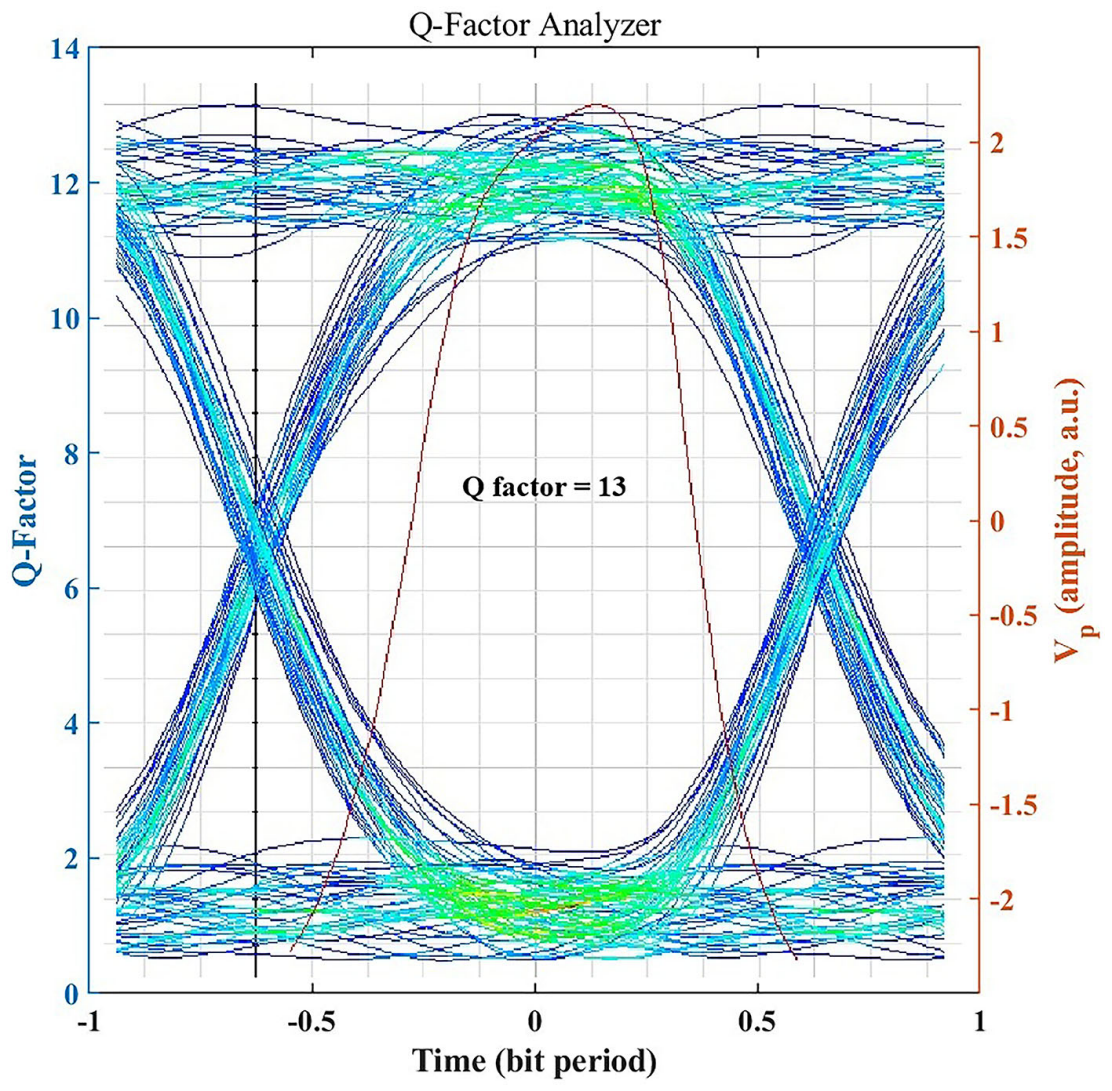
Eye diagram and Q factor under clear-air conditions.

The Q-factor provides a quantitative measure of signal integrity by expressing the separation between logic 1 and 0 levels relative to noise. Figure 4 illustrates an achieved Q-factor of ~13 for the clear-air 1.25 Gbps link, which is well above the typical threshold Q ≈ 6 (corresponding to BER = 10 ^−9^) required for robust, low-error optical communication. A Q-factor of this magnitude indicates a substantial noise margin, indicating strong analytical link margin under the assumed atmospheric and alignment conditions. Even under seasonal haze or light rain, the system maintains Q > 6 (as shown later), underscoring its reliability and resilience to weather-induced impairments. All BER values corresponding to high Q-factor (Q > 6) are reported as BER < 10 ^−12^ to reflect practical measurement limits.

### BER degradation with link distance

**Figure 5.**
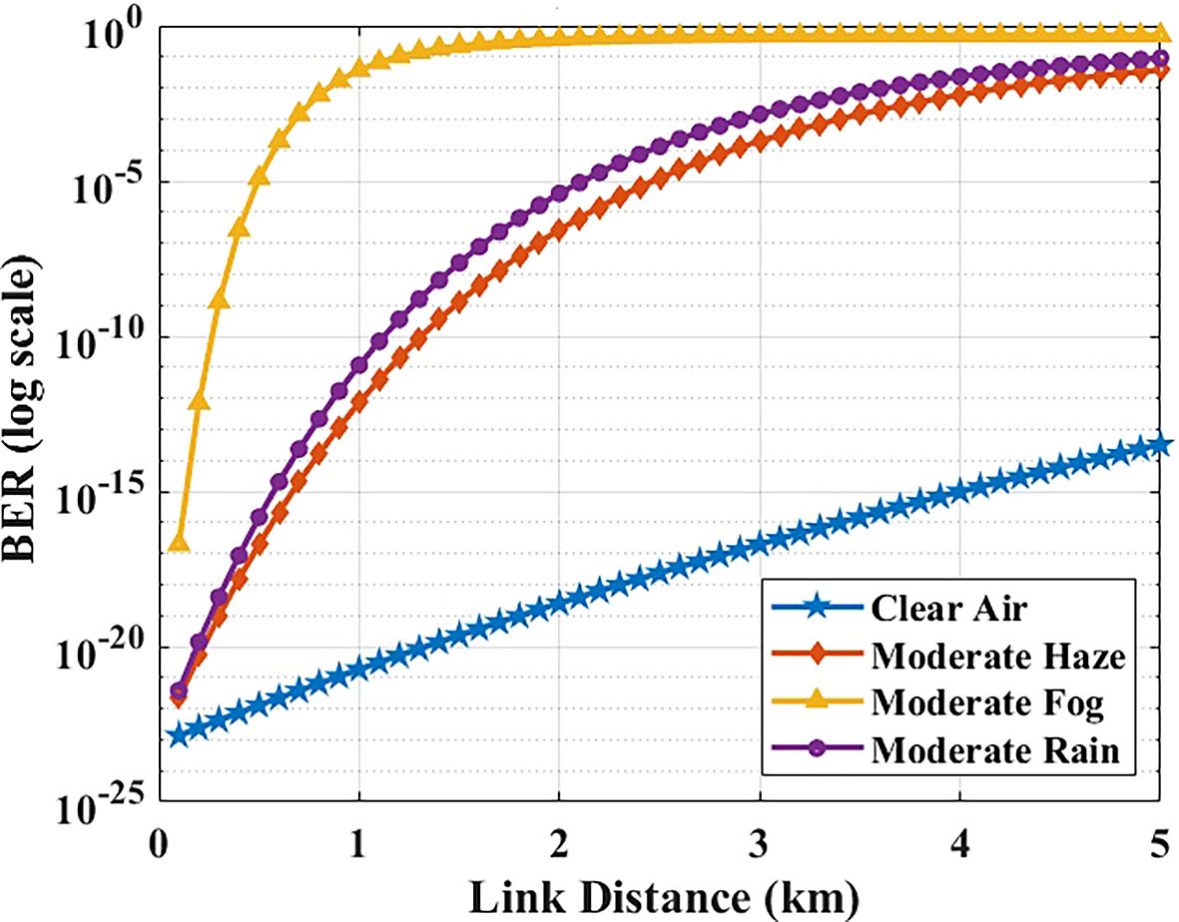
BER versus link distance in different weather conditions.

The relationship between link distance and BER was examined via MATLAB simulations. Figure 5 plots the BER as a function of transmission distance in different weather conditions. As expected, increasing the distance causes the received optical power to drop (due to path loss and attenuation), which in turn degrades the BER. The system predicts BER < 10 ^−9^ up to about 2 km, within the modeled atmospheric and alignment assumptions. Beyond 2 km, the BER begins approaching the 10 ^−9^ threshold under these weather conditions. Most inter-building distances in Maltese hospital campuses are within 1–2 km, indicating that the evaluated configuration provides analytical margin within the explored parameter space. Even at 5 km (the extreme evaluated range), the BER remains below 10 ^−9^ in clear/hazy conditions. Under moderate rain, BER increases to ~10 ^−8^, which slightly exceeds the strict error-free threshold but can be effectively mitigated with forward error correction (FEC). This ensures that reliable and secure data transfer is still achievable, even at extended distances, for hospital communication requirements.

### Received power vs. link distance

**Figure 6.**
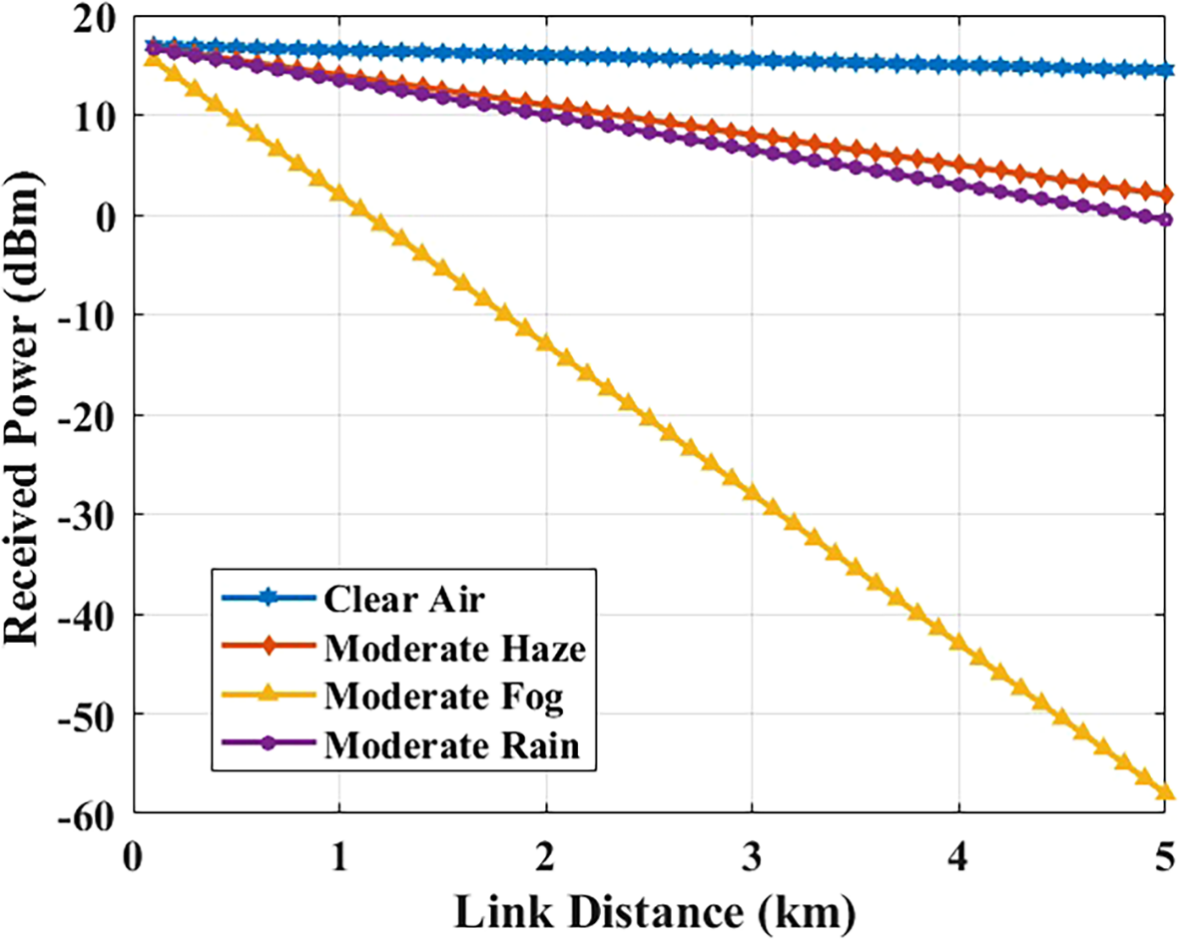
Received optical power versus link distance in different weather conditions.

The distance between transmitter and receiver results in decreased received optical power through free-space path loss, combined with weather-based attenuation effects. Figure 6 shows the received power for different link lengths under different weather conditions (3.5 dB/km, representative of Malta’s climate).

At a link distance of 1 km, the received power remains at approximately −6.5 dBm, which is well above the receiver sensitivity threshold and thus enables reliable optical signal conversion. It means that, assuming the attenuation model, the considered configuration has enough analytical power margin in standard hospital campus distances (12 km).

The possible FSO link distance is dependent on both BER degradation and receiver sensitivity. While the BER can be acceptable at larger distances with a clear atmosphere, reliable operation also requires that the received optical power is above the receiver sensitivity threshold of −35 dBm. When the weather is rainy and there is haze, the received power will progressively be reduced, thus bringing the operational range down. In view of this, the following practical deployment scenarios are recommended to ensure the link margin with respect to various weather conditions within the analytically validated distance range.

### Link availability under maltese weather conditions

The reliability assessment of FSO systems heavily depends on link availability measurements, especially when used in critical applications like hospital communications, where minimal signal interruptions disrupt data integrity and real-time monitoring functions.

**Figure 7.**
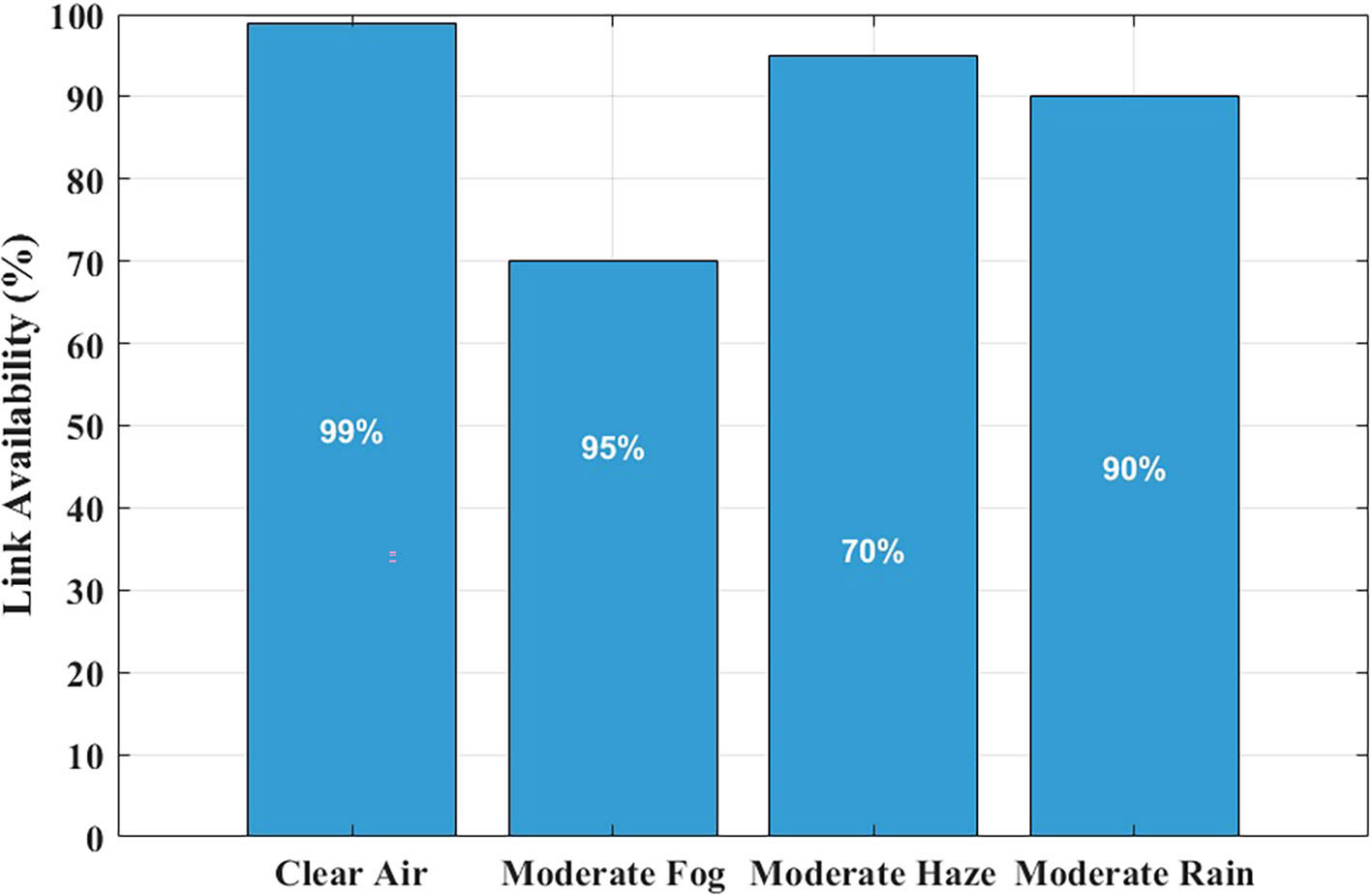
Link availability under Maltese weather conditions.

The link availability of the system is illustrated in Figure 7 for four distinct Maltese weather conditions, assimilating visibility information of clear air, haze, fog, and rain conditions.

Link availability means the percentage of time the FSO system is able to have a consistent connection. This value is estimated in this study by verifying that the received signal power remains above the receiver sensitivity threshold of −35 dBm, with a link margin of ~29 dB under clear-air conditions. With MATLAB, values of attenuation as a result of the effects of weather (e.g., haze, rain, and fog) based on the visibility are used to estimate the received power. When the signal exceeds the threshold, it is said that the link is available. The cumulative time of such conditions is finally divided by the total time of observation to arrive at the percentage of availability of links.

Communication links under clear-air conditions exhibit near-perfect availability, reflecting high predicted availability under the modeled conditions. The system maintains ~99% availability under moderate rain and light haze. The link availability drops below 85% when moderate fog conditions with their high attenuation levels occur. However, fog events in Malta are rare—less than two days per year—making their impact limited in the statistical climate model used in this study. This highlights that reliable hospital-grade connectivity can be achieved without hybrid RF backup, as rare fog events can be managed through automatic power control or adaptive data-rate reduction, rather than costly RF redundancy.

### BER performance under Maltese weather conditions

**Figure 8.**
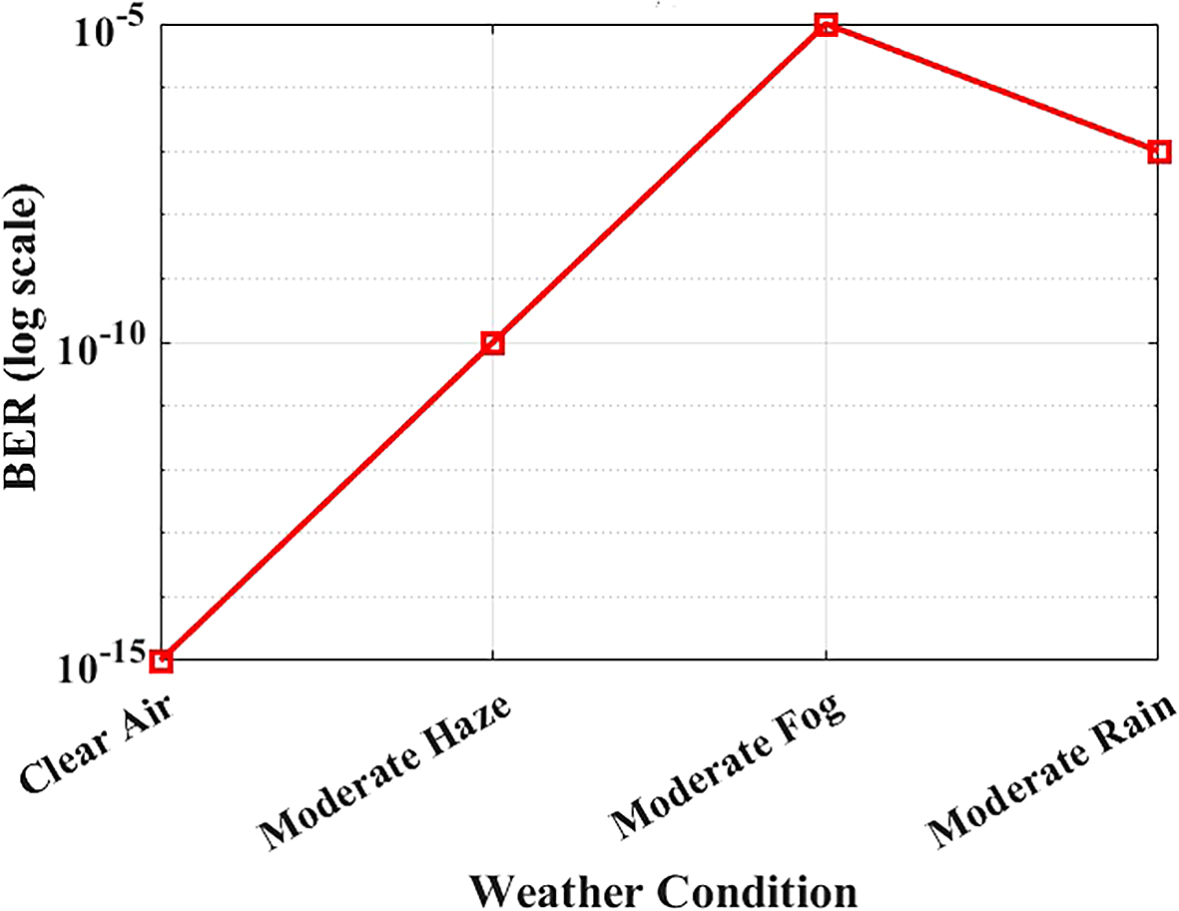
BER performance under different Maltese weather conditions.

The reliability of optical communication systems depends heavily on the Bit Error Rate (BER), a key parameter used to assess transmission accuracy. Figure 8 illustrates how BER varies under different weather conditions using attenuation values derived from actual visibility data in Malta. The system was simulated at a bit rate of 1.25 Gbps over a fixed link distance of 1 km, using attenuation levels specific to each weather scenario: 0.43 dB/km for clear air, 2.7 dB/km for moderate haze, 3.5 dB/km for moderate rain, and 15 dB/km for moderate fog.

Under clear-air conditions, the BER is effectively near zero, indicating operation within the practical error-free simulation region. Reported BER values for clear-air, haze, and rain conditions remain in the practical error-free region (BER < 10 ^−12^). Moderate fog causes extreme attenuation and results in significant degradation, yet the system maintains operational functionality, albeit with reduced performance. Haze and rain cause moderate performance loss due to combined scattering and absorption effects, but the degradation remains within acceptable limits. Nevertheless, the FSO system predicts BER < 10 ^−9^ under clear-air, haze, and moderate rain conditions within the deterministic attenuation model used in this study.

### Q-Factor as a measure of signal integrity

**Figure 9.**
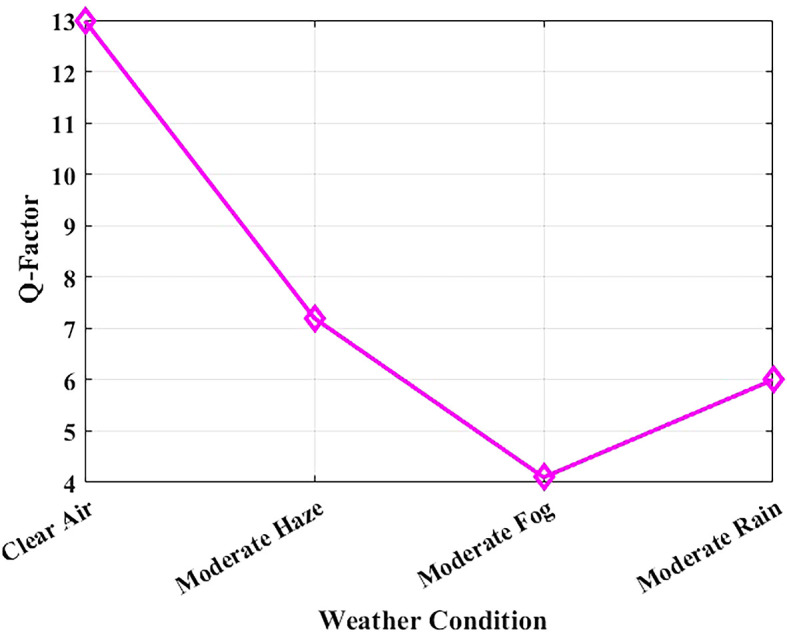
Q-factor variation with weather conditions in Malta.

The Q-factor relates directly to Bit Error Rate (BER) and to the signal-to-noise ratio (SNR) since it measures signal clarity. Deterioration of the weather conditions results in increased atmospheric attenuation and reduced Q-factor, both of which can be observed in Figure 9. The system was simulated at a bit rate of 1.25 Gbps over a constant link distance of 1 km, with attenuation values derived from Maltese visibility data: clear air (0.43 dB/km), moderate haze (2.7 dB/km), moderate rain (3.5 dB/km), and moderate fog (15 dB/km).

With clear air conditions, the Q-factor is high at 12.99, which implies good signal integrity. In moderate haze and rainfall, the Q-factor remains above the critical threshold of 6, is to suggest that the simulated association is within the realistic low-error band (BER < 10 ^−9^) under the modeled assumptions. However, in foggy weather, where visibility is severely reduced by high attenuation, the Q-factor diminishes considerably. In such cases, power control adjustments or temporary data-rate reduction may be required to maintain performance, as it acknowledges that the attenuation due to fog is a worst-case scenario in the climate model. These outcomes indicate that the proposed FSO 1550 nm configuration can be analytically stable in standard Maltese weather conditions in the studied parameter space. Hospital communication services would, however, need additional field validation and service level analysis to be deployed in practice.

### Weather condition frequency and its impact on FSO attenuation

**Figure 10.**
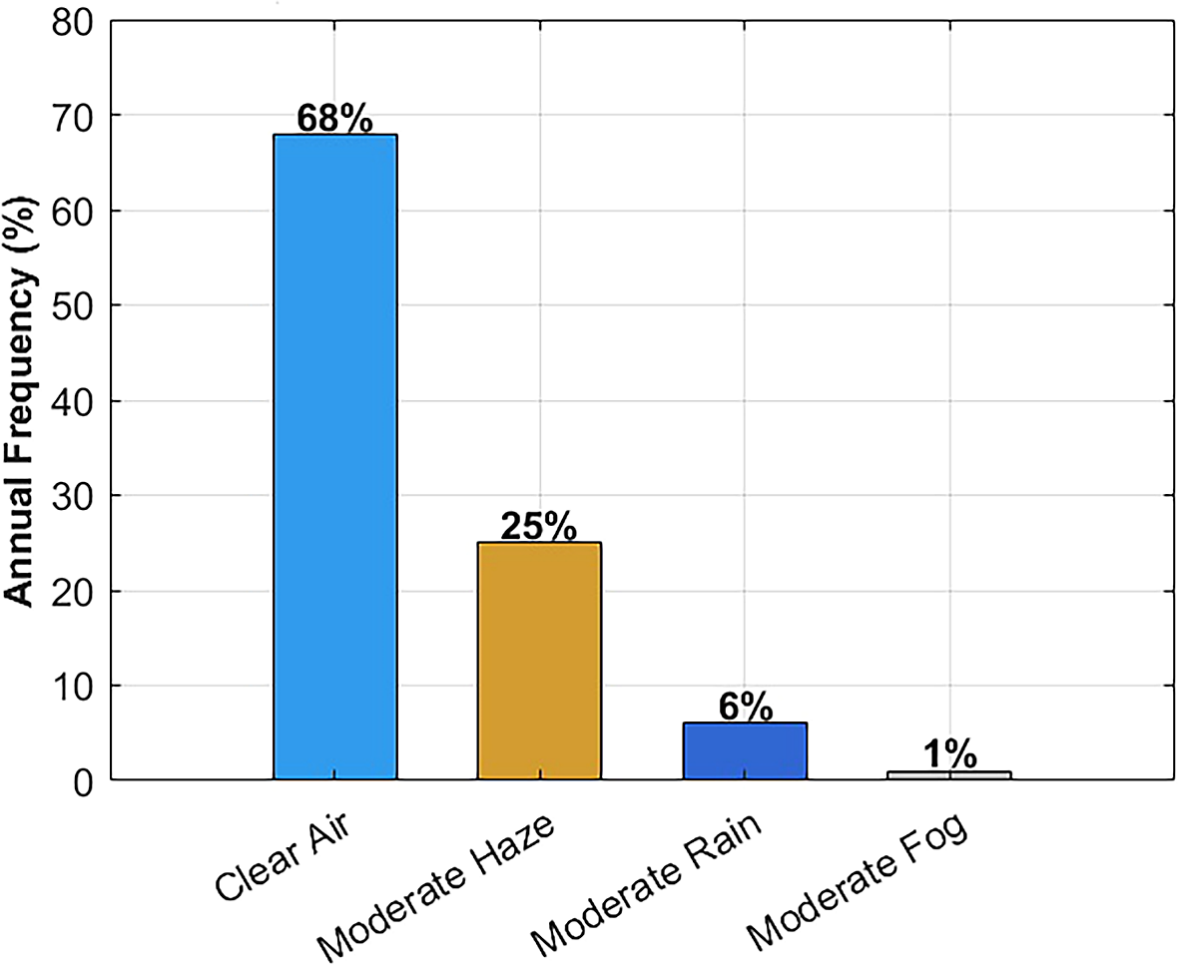
Annual frequency distribution of key weather conditions in Malta.

In order to guarantee the applicability of the simulation setting to the real weather patterns occurring in Malta, meteorological data obtained through official sources are considered over the period 2020–2023. ^21^ Figure 10 shows the frequency distribution of some major weather conditions that may affect FSO signal propagation. The annual climate picture is characterized mainly by the dominance of clear air, as it happens approximately 68% of the time, then moderate haze comes in second at 25%. The percentage of moderated rain is about 6%, and that of fog is so tiny to be seen, less than 1%.

These statistics support the modeling decision to prioritize clear-air, haze, and rain scenarios in the system’s attenuation modeling and performance testing. The rare occurrence of fog supports the use of a simplified, standalone FSO system without the need for hybrid RF redundancy in most cases, while still ensuring reliable hospital connectivity.

## Conclusion

This research presents a regionally optimized Free-Space Optical (FSO) communication system analytically evaluated for hospital inter-departmental networks in Malta. The system offers an EMI-free alternative to conventional RF-based solutions within the modeled assumptions by eliminating electromagnetic interference (EMI) risks in medical environments while, demonstrating favorable simulated performance under Mediterranean weather conditions.

The architecture, based on a 1550 nm WDM-based transmitter and sensitive PIN photodiode receiver, was simulated under realistic climate scenarios using OptiSystem 21 and MATLAB R2024b.

Signal performance showed operation within the practical error-free simulation region (BER < 10 ^−12^ and a Q-factor of 13 in clear-air conditions. The system predicts Q-factor values above 6, and BER metrics below 10 ^−9^ when operating in conditions with moderate rain and haze since Q-factor values exceed 6 and BER metrics remain under 10 ^−9^. Link availability exceeds 99% under clear-air and moderate haze conditions except for the rare occurrences of fog that affect Malta fewer than two days annually. Received power measurements indicate signal integrity maintenance at distances up to 5 kilometres, while operation reaches its best state at distances less than 2 kilometres, which fulfils all potential hospital deployment needs.

This study indicates that the suggested FSO design is capable of analytically supporting regional attenuation conditions such as coastal haze and seasonal rain, while also ensuring EMI resistance, simplified installation, and reduced deployment costs. The decision about hybrid redundancy, nevertheless, is to be made based on the requirements of institutional reliability and targets of service levels.

Overall, this research delivers a hospital-oriented FSO communication framework based on simulation. Tailored for Mediterranean climates with limited fog occurrences. The proposed system predicts high-speed optical link feasibility for critical medical applications such as real-time monitoring, medical imaging, and electronic health records, while supporting scalable, future-ready healthcare networking infrastructures.

Before it can be implemented in the real hospital setting, further experimental validation and long-term field measurements are needed.

## Future work and recommendations

Although the current system functions well under Malta’s current climate conditions, a lightweight backup system that combines FSO and RF technology could serve as a redundancy measure to extend reliability across areas with changing climates. Conducting field trials on actual hospital campuses is recommended to validate simulation results, assess real-world performance, and identify potential installation challenges. Future applications of FSO systems include combining with 6G networks and Internet of Medical Things devices to aid in launching smart hospitals that support real-time data exchange, advanced diagnostics, and seamless inter-departmental communication.

## Ethics and consent

Ethical approval and consent were not required for this study as it did not involve human participants, animals, or sensitive personal data.

## Data Availability

All data underlying this study are available from the Zenodo repository FSO Communication System in Maltese Hospitals ( https://doi.org/10.5281/zenodo.17136324). The repository contains the following underlying data: Malta_Weather_Conditions.csv (weather condition data including visibility, attenuation, and annual frequency derived from Malta Airport observations and NSO climate statistics), Malta_Monthly_Averages.csv (monthly climate averages including temperature, sunshine hours, and rainfall obtained from the NSO Climate Publication 2022), and Simulation_Output_FSO.xlsx (Q-factor, BER, and received optical power results generated using OptiSystem and MATLAB simulations). The repository also includes extended data comprising Supplementary Table 1, which presents the complete weather–climate dataset used as input for simulation models, and Supplementary Figure Legends, which provide the titles and descriptions of all figures used in the manuscript. All data are available under the terms of the Creative Commons Zero “No rights reserved” data waiver (CC0 1.0 Public domain dedication).
