# Supplementary material for: Design and optimization of a Free-Space Optical (FSO) communication system for reliable outdoor connectivity in hospital departments in Malta
Source: Open Res Eur. 2026 May 29;5:311. Originally published 2025 Oct 15. [Version 4] doi: 10.12688/openreseurope.21257.4 (PMC13146486; doi:10.12688/openreseurope.21257.4)
Supplement: Peer review history

# Reviewer report: Reviewer response for version 3

**DOI:** 10.21956/openreseurope.25479.r72709

**Published:** 2026-05-20

**Authors:** Martyn Fice

**Affiliations:** 1 University College London, London, UK

The paper describes simulations of the performance of a free-space optical (FSO) communications link under different weather conditions. The communications link performance is calculated using a straightforward model based on well-known equations. The paper's value lies in applying this model to the particular weather conditions of Malta and in the specific scenario of short-range (up to a few kilometres) links within a hospital campus, therefore showing that there is at least a reasonable expectation that a working system could be implemented with satisfactory performance under these conditions.

 In my opinion, there are a number of aspects of the paper that need addressing before it would be suitable for indexing.

1. The communications link model used does not include several important factors that present problems for FSO communications in practical, outdoor implementations. The most important of these is atmospheric turbulence. There should be some consideration of this in the specific case of a system operating under the atmospheric conditions prevalent in Malta or, if the authors have good reasons for neglecting its effects, they should explain why this is the case. Beam pointing accuracy and movement of outdoor transmitter and receiver units will give rise to increased worst-case link losses, even in clear-air conditions, and these should be included or at least discussed.
2. Some aspects of the FSO channel model described on p. 5 are confusing or incorrect and these should be checked and corrected as necessary. (a) The units of alpha in Eq. 3 should be given. (b) The authors should explain how the beam divergence of 1.5 mrad is obtained. This is much larger than the diffraction-limited divergence for an aperture of 10 cm diameter, so the assumptions made when obtaining this value should be explained (that is, summarise the conditions given in Ref. 9, which is not freely available). (c) The numerical result in Equation 6 is incorrect; using the numerical values stated, I calculate the loss to be 17.5 dB. If this changes later calculations, those should also be amended.
3. The methodology for calculating the link availability shown in Figure 7 (and stated elsewhere in the paper) should be explained. Given the excellent performance of the simulated system in clear-air conditions, I do not understand why the link availability for these conditions is not 100%. Presumably the value of 99% is obtained from the statistical distribution of observed visibility under nominally clear air conditions, but this is not clearly explained. There is an error in labelling the bars in Figure 7 (the link availability for Moderate Fog is clearly not 95%, and that for Moderate Haze is not 70%).
4. There are discrepancies between the text and the values shown in Figure 5 and 6. For instance, at 1 km link distance, the text states that the received power is -6.5 dBm. It is not stated which weather condition this result relates to (this should be stated), but for all four weather conditions in Figure 6 the received power is at least 0 dBm. There are similar problems in the discussion of the BER in Figure 5. There are also discrepancies between Figures 8 and 9: the Q factor for moderate rain is around 6 (Figure 9), but the BER in Figure 8 is much larger than the 1E-9 corresponding to this value of Q.
5. Thermal noise is usually higher than that given by Eq. 14, because of the noise figure of the receiver front end. Although shot noise may dominate in clear-air conditions, thermal noise will be more significant when channel losses are higher, and therefore the receiver noise figure should be considered.
6. To account for practical implementation, additional system margin (additional fixed system losses) might be usefully included.
7. The Introduction focuses too much on the papers by the authors. Works by other authors should be included.

Is the study design appropriate and does the work have academic merit?

Yes

Is the work clearly and accurately presented and does it cite the current literature?

Partly

If applicable, is the statistical analysis and its interpretation appropriate?

Yes

Are all the source data underlying the results available to ensure full reproducibility?

Yes

Are the conclusions drawn adequately supported by the results?

Yes

Are sufficient details of methods and analysis provided to allow replication by others?

Partly

Reviewer Expertise:

Photonics; optical and wireless communications.

I confirm that I have read this submission and believe that I have an appropriate level of expertise to confirm that it is of an acceptable scientific standard, however I have significant reservations, as outlined above.

# Reviewer report: Reviewer response for version 3

**DOI:** 10.21956/openreseurope.25479.r72714

**Published:** 2026-05-05

**Authors:** Yus Natali

**Affiliations:** 1 Telkom University, Jawa Barat, Indonesia

**The paper title is “Design and optimization of a Free-Space Optical (FSO) communication system for reliable outdoor connectivity in hospital departments in Malta”.**

 he manuscript presents a simulation-based design of an FSO communication system tailored for hospital environments in Malta. While the topic is relevant and the modeling framework is clearly structured, several critical issues limit its suitability for a publication in this journal.

 First, the term “optimization” is misleading, as the study only performs parametric evaluation and does not employ any formal optimization technique. Second, the absence of statistical modeling, such as Monte Carlo simulation or turbulence analysis, significantly limits the reliability of the results in realistic FSO environments. Third, the work lacks experimental validation or benchmarking with existing systems.

 Furthermore, the conclusions tend to overstate the real-world applicability of the proposed system, despite being purely simulation-based. The channel model also does not consider key impairments such as atmospheric turbulence, pointing errors, or scintillation effects.

 Therefore, major revisions are required before this manuscript can be considered for publication in this journal. Please revise the paper as follows:

 1. Clarification of Figure 1 and Link Configuration

 The authors are requested to provide a more detailed explanation of Figure 1, particularly by clearly identifying the roles of each node as either transmitter or receiver. For clarity, it is recommended to include illustrative examples, such as the Radiology Block acting as transmitter (A), the Main Hospital Block as receiver (B), the Main Hospital Block as transmitter (C), and the Administration & Records Data Block as receiver (D).

 Furthermore, it is necessary to clarify whether all possible links between these nodes have been considered in the simulation. The manuscript should explicitly state the actual distances between the buildings, as this information is essential for validating the feasibility of the proposed FSO links.

 In addition, the authors are encouraged to analyze different propagation scenarios. For instance, under clear weather conditions, it should be demonstrated whether a direct single-hop link from the Radiology Block (A) to the Administration & Records Data Block (D) is feasible. Conversely, under adverse weather conditions such as fog, a multi-hop configuration (e.g., A–B and C–D) may be required. Therefore, the manuscript should include a comparative analysis of single-hop and multi-hop communication, accounting for realistic inter-building distances and atmospheric attenuation.

 2. Justification of BER Threshold

 The authors have adopted a BER threshold of less than 10−12. Please provide appropriate references to justify the selection of this value, particularly in the context of practical optical communication systems and accepted performance standards.

 3. Revision of Figure Presentation

 The naming and presentation of graphs in Figures 3–10 should be revised to conform to standard academic formatting. Specifically, redundant titles (e.g., “BER vs. Link Distance”) placed above the figures should be removed. Instead, all descriptions should be incorporated into the figure captions to maintain consistency with journal guidelines.

 4. Clarification of Link Availability Analysis

 The methodology used to evaluate link availability in the FSO system is not sufficiently explained. The authors are requested to clearly describe the analytical approach used to determine link availability, including the criteria and thresholds applied. In particular, it is important to specify whether any established standards or benchmarks (e.g., ITU recommendations or availability targets such as 99.9%) have been adopted.

 5. Relationship Between BER, Receiver Sensitivity, and Link Distance

 The analysis of link distance based solely on BER is insufficient without considering receiver sensitivity. Given that the receiver sensitivity is specified as −35 dBm, the achievable link distance will depend on whether the received power remains above this threshold under varying weather conditions.

 Therefore, the authors are encouraged to integrate BER analysis with received-power considerations when determining the maximum link distance. This approach will enable a more realistic assessment of system performance for both single-hop and multi-hop configurations. Additionally, such an analysis would provide clearer insight into the system's operational limits under different atmospheric conditions.

Is the study design appropriate and does the work have academic merit?

Yes

Is the work clearly and accurately presented and does it cite the current literature?

Yes

If applicable, is the statistical analysis and its interpretation appropriate?

Partly

Are all the source data underlying the results available to ensure full reproducibility?

Partly

Are the conclusions drawn adequately supported by the results?

Partly

Are sufficient details of methods and analysis provided to allow replication by others?

Partly

Reviewer Expertise:

Wireless optical technology/communication, microwave photonics, optical antenna, and electro-optic

I confirm that I have read this submission and believe that I have an appropriate level of expertise to confirm that it is of an acceptable scientific standard, however I have significant reservations, as outlined above.

# Response

**Published:** 2026-05-16

**Authors:** Ajay Sharma

**Affiliations:** Computer Information Systems, University of Malta Faculty of Information and Communications Technology, Msida, Malta

The comments made by the reviewer are appreciated and gratefully accepted. Much appreciated were the suggestions, which contributed to a better clarity, technical interpretation, and presentation of the manuscript. The manuscript has been revised accordingly, with explanations of the symbolic representation of the hospital FSO topology, the selection of BER threshold, the explanation of link availability analysis, the introduction of receiver sensitivity considerations along with BER-based distance evaluation, and the modification of the applicability of the real-world deployment. Besides, the guidelines of the journal have been followed in the formatting of figures, and the limitations due to stochastic atmospheric effects and experimental validation have been clearly acknowledged in the revised manuscript.

**Clarification of Figure 1 and Link Configuration** **Comment 1:** *The authors are requested to provide a more detailed explanation of Figure 1, particularly by clearly identifying the roles of each node as either transmitter or receiver....* **Response:** The idea is very useful and appreciated. The symbolic interdepartmental FSO communication topology in the hospital is revised and clarified as shown in Figure 1. The updated figure represents conceptual communication nodes within the hospital, such as Administration (D), Emergency (B), Main Hospital (C) and Radiology (A). Some explanatory text has also been included after the discussion of Figure 1 to clarify that the figure does not represent a specific network architecture but rather represents possible paths of inter-building communication via FSO. In the revised manuscript, the range of simulated link distances is clearly specified as between 0.5 km and 2 km, which is typical of current hospital campus deployments in Malta. In addition, a discussion on possible single-hop and multi-hop communication scenarios under various atmospheric conditions is provided. The manuscript now elaborates that, under clear weather conditions, longer single-hop links might be possible, and shorter multi-hop links might offer better reliability in adverse conditions like fog. **Justification of BER Threshold** **Comment 2:** *The authors have adopted a BER threshold of less than 10−12. Please provide appropriate references...* **Response:** The manuscript has been revised to give more reasons as to why the practical operating region of optical communication systems has been chosen to be BER < 10 ^−12^. The BER analysis section is supplemented with supporting discussion and reference to the standard optical communication literature. The revised text has been made clearer that in practice, BER values lower than 10 ^−12^ are regarded as near-error-free, since the further reduction of BER has little significant impact on the reliability of the optical communication system. **Revision of Figure Presentation** **Comment 3:** *The naming and presentation of graphs in Figures 3–10 should be revised...* **Response:** Figures 3–10 have been presented in a new format based on journal formatting. Previously, the title of the graph was on display above the graph; this has been removed, and all descriptions for the figure are now in the figure caption so that they are consistent throughout the manuscript. **Clarification of Link Availability Analysis** **Comment 4:** *The methodology used to evaluate link availability....* **Response:** We concur that it is a simulation study with no field experiments. The updated manuscript now clearly mentions this limitation and suggests that further field tests in hospital campuses should be done to determine performance under operational conditions. Moderating claims of deployment readiness has been done. **Relationship Between BER, Receiver Sensitivity, and Link Distance** **Comment 5:** *The analysis of link distance based solely on BER is insufficient....* **Response:** The manuscript has been revised so as to clarify the methodology adopted for evaluating the availability of a link under different atmospheric conditions. The "reliable link operation" assumption is explained, as both BER conditions and receiver sensitivity were true. Specifically, the availability of the link was assessed by keeping BER below acceptable operational limits whilst at the same time keeping the received optical power above the receiver threshold sensitivity of -35 dBm. The updated text also makes it clear that the analysis is based on the standard telecommunication reliability practices for a high-availability optical communications system. **Comment on Statistical Modeling / Experimental Validation** **Comment 6:** *The absence of statistical modeling, turbulence analysis, and experimental validation limits realism.* **Response:** The main purpose of the present work was the development of a deterministic simulation-based model for the evaluation of FSO performance in Maltese atmospheric conditions. In order to overcome this deficiency, an explicit discussion has now been incorporated in the manuscript to acknowledge the fact that advanced effects like scintillation, beam wander, pointing errors, and atmospheric turbulence have not been considered in this study and will be the subject of future study in the statistical and experimental analysis. **Comment on Real-World Applicability** **Comment 7:** *Conclusions overstate practical applicability.* **Response:** The conclusion section has been updated to tone down some of the sentences about practical deployment and applicability in practice. The revised manuscript now focuses on the results presented, showing that the proposed FSO architecture could potentially be a viable solution for deployment under modeled Maltese atmospheric conditions, but does not assert guaranteed deployment performance.

# Reviewer report: Reviewer response for version 2

**DOI:** 10.21956/openreseurope.23814.r67809

**Published:** 2026-02-24

**Authors:** Amit Rathi

**Affiliations:** 1 Manipal University Jaipur, Jaipur, Rajasthan, India

**Summary of the article**

- The article proposes a 1550 nm Free-Space Optical (FSO) communication system for hospital connectivity in Malta, using OptiSystem and MATLAB simulations.
- It evaluates BER, Q-factor, received power, and link availability under local weather conditions.
- Results suggest reliable performance up to 2 km and high availability (>99%) under clear air, haze, and rain.
  65b80e80-0823-4fe7-9cc6-705e848…

**Strengths**

- Clear structure with relevant mathematical modelling and simulation framework.
- Uses real Maltese climate data, improving practical relevance.
- Literature references are generally current and appropriate.
- Data availability via repository supports transparency.

**Weaknesses (must address)**

- No statistical analysis, uncertainty modelling, or Monte Carlo simulation.
- Methodology lacks sufficient simulation details for full replication.
- “Optimization” is claimed but not systematically demonstrated.
- No experimental validation or comparison with existing deployed systems.
- Conclusions overstate real-world applicability and cost benefits.

**Required improvements**

- Add statistical and sensitivity analysis.
- Provide full simulation parameters and implementation details.
- Clarify novelty and revise conclusions to reflect simulation-based findings only.

Is the study design appropriate and does the work have academic merit?

Partly

Is the work clearly and accurately presented and does it cite the current literature?

Partly

If applicable, is the statistical analysis and its interpretation appropriate?

No

Are all the source data underlying the results available to ensure full reproducibility?

Partly

Are the conclusions drawn adequately supported by the results?

Partly

Are sufficient details of methods and analysis provided to allow replication by others?

Partly

Reviewer Expertise:

Microstrip Antenna, Optical Material, optoelectronics

I confirm that I have read this submission and believe that I have an appropriate level of expertise to confirm that it is of an acceptable scientific standard, however I have significant reservations, as outlined above.

# Response

**Published:** 2026-03-26

**Authors:** Ajay Sharma

**Affiliations:** Computer Information Systems, University of Malta Faculty of Information and Communications Technology, Msida, Malta

We thank the reviewer very much for the thorough and constructive review. The remarks have done a great deal in enhancing the clarity, rigour, and placement of the manuscript. Each of these areas is discussed below.

**Comment 1:** *No statistical analysis, uncertainty modelling, or Monte Carlo simulation.* **Response:** We recognize that the current study utilizes deterministic modeling of links and attenuation over atmospheric turbulence challenges instead of stochastic Monte Carlo-based atmospheric turbulence simulations. This limitation has been corrected in the manuscript, which now clearly mentions this limitation in the Methodology and Conclusion sections. Statistical reliability claims have been toned down, and findings have now been clearly presented as a simulation-based analytical feasibility under specified attenuation assumptions.

**Comment 2:** *Methodology lacks sufficient simulation details for full replication.*

**Response:** The methodology section has been extended to include full parameters of simulation and modeling assumptions. Wavelength (1550 nm), transmitter power (50 mW), extinction ratio (20 dB), beam divergence (1.5 mrad), transmitter aperture (10 cm), receiver aperture (20 cm), photodiode responsivity (0.8 A/W), bandwidth (0.94 GHz), receiver sensitivity ( -35 dBm) and temperature (300 K) now have explicit values. The mathematical attenuation, geometric loss, noise modeling and BER estimation equations are well documented. The manuscript has now been altered to indicate that replication may be done with OptiSystem or similar numerical solvers using the equations provided.

**Comment 3:** *“Optimization” is claimed but not systematically demonstrated.*

**Response:** Optimization has been explained throughout the manuscript. The paper does not use an explicit nonlinear optimization problem. Rather, the parametric evaluation was organized and conducted over attenuation levels, the link distance, and sensitivity constraints on the receiver. The manuscript now outlines the method as parameter-tuned analytical analysis instead of algorithmic global optimization.

**Comment 4:** *No experimental validation or comparison with existing deployed systems.* **Response:** We concur that it is a simulation study with no field experiments. The updated manuscript now clearly mentions this limitation and suggests that further field tests in hospital campuses should be done to determine performance under operational conditions. Moderating claims of deployment readiness has been done.

**Comment 5:** *Conclusions overstate real-world applicability and cost benefits.*

**Response:** The Conclusion part has been updated significantly. Claims about guaranteed deployment performance, the absence of hybrid redundancy, and guaranteed cost superiority have been toned down. The conclusions now indicate the feasibility of simulations under the modeled climate assumptions and clearly indicate that the practical implementation would need additional validation and institutional assessment.

**Comment 6:** *Add statistical and sensitivity analysis.*

**Response:** Although a complete Monte Carlo turbulence model was not used, the manuscript now also indicates the deterministic character of the modeling and explains that the results are attenuation-limited cases based on actual meteorological observations. Stochastic modeling and analysis of uncertainty have become the next step in work recommendations for the future.

**Comment 7:** *Provide full simulation parameters and implementation details.*

**Response:** All the assumptions of the simulation parameters and the environmental parameters are now clearly stated in the Methodology section to allow independent replication.

**Comment 8:** *Clarify novelty and revise conclusions to reflect simulation-based findings only.* **Response:** The novelty statement is now revised to highlight the combination of Malta-specific climate modeling, 1550 nm wavelength selection, and hospital EMI constraints in a single deterministic model. The conclusions have now been updated to clearly point out that the results are simulated and reflect analytical capability, more than field-validated deployment performance.

**Evaluation Questions**

**Comment *:*** *Is the work clearly and accurately presented, and does it cite the current literature? – Partly*

**Response:** The literature review has been streamlined to enhance positioning, as well as eliminate exaggerated statements. The text has become more inclusive of the difference between simulation results and real-world implementation issues.

**Comment:** *Is the study design appropriate, and does the work have academic merit? – Partly* **Response: **It has enhanced the methodological transparency and made deterministic assumptions clear to improve academic rigor and transparency.

**Comment:** *Are sufficient details of methods and analysis provided to allow replication by others? – Partly*

**Response: **Every modeling equation and system parameter has been completely defined to enable the re-creation with similar tools.

**Comment:** *Are sufficient details of methods and analysis provided to allow replication by others? – Partly*

**Response: **The fact that no probabilistic statistical analysis has been conducted and results are obtained through deterministic link-budget modeling is now expressly stated in the manuscript.

**Comment:** *Are all the source data underlying the results available to ensure full reproducibility? – Partly*

**Response: **The attenuation values are calculated based on publicly available Maltese meteorological datasets (2020–2023), and a reference is made. Equations and parameter assumptions in modeling are completely documented.

**Comment:** *Are the conclusions drawn adequately supported by the results? – Partly* **Response: **The conclusion has been changed to further concur with the findings of the simulations. Claims and cost guarantees at the deployment level have been eliminated or toned down.   We value the reviewer's critical analysis. The updates have enhanced the methodological transparency, as well as guaranteed that conclusions are put into perspective within the framework of the simulation-based analysis.

# Reviewer report: Reviewer response for version 2

**DOI:** 10.21956/openreseurope.23814.r64849

**Published:** 2025-12-11

**Authors:** Moustafa Hussein Aly

**Affiliations:** 1 Arab Academy for Science Technology and Maritime Transport, Alexandria, Alexandria Governorate, Egypt

Thank you for considering my comments.

 From my side, no more action is required.

 ((Indexed)).

Is the study design appropriate and does the work have academic merit?

Yes

Is the work clearly and accurately presented and does it cite the current literature?

Yes

If applicable, is the statistical analysis and its interpretation appropriate?

Partly

Are all the source data underlying the results available to ensure full reproducibility?

Partly

Are the conclusions drawn adequately supported by the results?

Partly

Are sufficient details of methods and analysis provided to allow replication by others?

Yes

Reviewer Expertise:

Optical Communications.

I confirm that I have read this submission and believe that I have an appropriate level of expertise to confirm that it is of an acceptable scientific standard.

# Reviewer report: Reviewer response for version 1

**DOI:** 10.21956/openreseurope.22991.r62753

**Published:** 2025-11-03

**Authors:** Moustafa Hussein Aly

**Affiliations:** 1 Arab Academy for Science Technology and Maritime Transport, Alexandria, Alexandria Governorate, Egypt

Thank you for your effort.

 I have some comments that must be considered in the revised manuscript.

 1) The equations and proposal are familiar.

 2) The values of the Q-factor are accepted and practical.

 3) The values of BER are wrong. Practically, all values below 10^-12 are wrong and not practical values. I think authors used cracked version of the program. 4) Use the equations to calculate the correct values of BER.

 4) References are up-todate.

Is the study design appropriate and does the work have academic merit?

Yes

Is the work clearly and accurately presented and does it cite the current literature?

Yes

If applicable, is the statistical analysis and its interpretation appropriate?

Partly

Are all the source data underlying the results available to ensure full reproducibility?

Partly

Are the conclusions drawn adequately supported by the results?

Partly

Are sufficient details of methods and analysis provided to allow replication by others?

Yes

Reviewer Expertise:

Optical Communications.

I confirm that I have read this submission and believe that I have an appropriate level of expertise to confirm that it is of an acceptable scientific standard, however I have significant reservations, as outlined above.

# Response

**Published:** 2025-11-21

**Authors:** Ajay Sharma

**Affiliations:** Computer Information Systems, University of Malta Faculty of Information and Communications Technology, Msida, Malta

We greatly appreciate the reviewer and his critical assessment and positive remarks. The feedback also enabled us to strengthen the technical clarity of the manuscript and align the reported results with the real-world standards of optical communication. Each suggestion has been properly considered, and the manuscript has been changed according to it.

**Comment 1:** *The equations and proposal are familiar.*

**Response:** We appreciate this observation. The proposed work develops the already existing models of optical communication and uses them in a new setting, EMI-free interdepartmental connectivity in Maltese hospitals. The contribution is the modification of the current FSO mathematical models to genuine Maltese Mediterranean meteorological data and to the healthcare setting that is regulated by the EMI safety standards (IEC 60601-1-2).

**Comment 2:** *The values of the Q-factor are accepted and practical.*

**Response:** The values of the Q-factor used in the manuscript were checked and do not exceed the range of practical values of the Q-factor in optical communication systems in real-life (Q > 6, which is the value of BER = 10 ^-9^ or less).

**Comment 3:** *The values of BER are wrong. Practically, all values below 10⁻¹² are wrong and not practical values. I think the authors used a cracked version of the program. Use the equations to calculate the correct values of BER.*

**Response:** This suggestion has been taken into consideration to correct the manuscript.

1. **Source of the issue:**

The numerically reported values of BER (e.g., 7.14 × 10 ^-39^) were a by-product of automatic mapping of the values of Q-factor versus BER provided by OptiSystem, based on the equation.  For a Q-factor of about 13, this equation yields a theoretical BER of ≈ 10⁻³⁹, which is not physically measurable.

1. **Corrections made:**

- Any occurrence of such values has been substituted with **BER < 10 ^-12^ (error-free region),** which is more realistic in terms of measurement limits.
- The above equation will be included in a new subsection named **Relation between Q-Factor and BER,** with a description that the values that are less than 10 ^-12^ are theoretical, and these values depict the error-free operating region.
- It has been noted that all the simulations were performed with the help of the **OptiSystem 21 (licensed version, University of Malta)** and **MATLAB R2024b.**

 

1. Outcome:

The obtained Q-factor results verify that the system works in the error-free regime with clear air, haze, and moderate rain, and it is physically realistic.

**Comment 4:** *References are up-to-date.*

**Response:** We are glad to hear this good comment. The reference list is checked and up to date, with the inclusion of publications made since 2020.

**Additional Review Points** **Review Criterion:** **Reviewer’s Observation:** **Author Response**  

- Clarity and accuracy: Yes: The updated version is more efficient as it differentiates the theoretical and practical BER interpretations.
- Study design and merit: Yes: No change required. The design is confirmed to be appropriate for applications of communication in hospitals.
- Methods and analysis: Yes: Additional clarification has been added on software licensing and BER calculation methodology. 
- Statistical analysis: Partly: The manuscript now includes an explicit Q–BER relationship and discussion of practical BER thresholds.
- Data reproducibility: Partly: the Zenodo dataset remains available and now includes detailed simulation outputs (OptiSystem and MATLAB).
- Conclusions supported by results: Partly: The new text has explicitly described the relationships between Q-factor and BER results and realistic system behaviour.
